# *Burkholderia pseudomallei* d‐alanine‐d‐alanine ligase; detailed characterisation and assessment of a potential antibiotic drug target

**DOI:** 10.1111/febs.14976

**Published:** 2019-07-16

**Authors:** Laura Díaz‐Sáez, Leah S. Torrie, Stuart P. McElroy, David Gray, William N. Hunter

**Affiliations:** ^1^ Division of Biological Chemistry and Drug Discovery School of Life Sciences University of Dundee UK; ^2^ Drug Discovery Unit Wellcome Centre for Anti‐Infectives Research School of Life Sciences University of Dundee UK; ^3^ European Screening Centre Newhouse, Biocity Scotland University of Dundee Newhouse UK; ^4^ Present address: BioAscent Discovery Ltd Bo'ness Road Newhouse Lanarkshire ML1 5UH UK

**Keywords:** antibacterial, enzyme assay, enzyme inhibition, peptidoglycan, target validation

## Abstract

*Burkholderia pseudomallei* is a serious, difficult to treat Gram‐negative pathogen and an increase in the occurrence of drug‐resistant strains has been detected. We have directed efforts to identify and to evaluate potential drug targets relevant to treatment of infection by *B. pseudomallei*. We have selected and characterised the essential enzyme d‐alanine‐d‐alanine ligase (*Bp*Ddl), required for the ATP‐assisted biosynthesis of a peptidoglycan precursor. A recombinant supply of protein supported high‐resolution crystallographic and biophysical studies with ligands (AMP and AMP+d‐Ala‐d‐Ala), and comparisons with orthologues enzymes suggest a ligand‐induced conformational change occurring that might be relevant to the catalytic cycle. The detailed biochemical characterisation of the enzyme, development and optimisation of ligand binding assays supported the search for novel inhibitors by screening of selected compound libraries. In a similar manner to that observed previously in other studies, we note a paucity of hits that are worth follow‐up and then in combination with a computational analysis of the active site, we conclude that this ligase represents a difficult target for drug discovery. Nevertheless, our reagents, protocols and data can underpin future efforts exploiting more diverse chemical libraries and structure‐based approaches.

## Graphical abstract

Enzymatic and structural characterisation of recombinant *Burkholderia pseudomallei* d‐alanine‐d‐alanine ligase, with the development and optimisation of ligand binding assays supported a search for novel inhibitors by screening of three compound libraries. The failure to find high value hits in combination with a computational analysis of the active site indicates this ligase represents a difficult target for drug discovery.

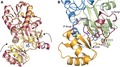

**Abbreviations**
BLI: biolayer interferometry
BSA: buried surface area
DCS: d‐cycloserine
Ddl: d‐alanine‐d‐alanine ligase
HTP: high throughput
PEP: phosphoenolpyruvate
PG: peptidoglycan
PK/LDH: phosphoglycerate kinase/lactate dehydrogenase

## Introduction

*Burkholderia pseudomallei,* a serious pathogen of humans and animals, causes melioidosis. This Gram‐negative bacterium is widely distributed in the environment and is classified as a Category B biowarfare agent by the U.S. Centers for Disease Control and Prevention (CDC, http://www.cdc.gov). Treatment of melioidosis involves intravenous high‐intensity application of cephalosporins for up to 2 weeks then an eradication phase to prevent recurrence that can involve the use of antifolate combinations for up to 20 weeks. This is an expensive treatment often complicated by side effects and a further issue is that strains resistant to the current drugs have been identified 1, 2. New antibacterial strategies are being considered 3 and to this end, we sought to investigate the potential of key enzymes as new therapeutic targets and to generate the data, reagents and protocols that support an assessment of these targets for early stage drug discovery.

The peptidoglycan (PG) layer contributes an important protective shield for Gram‐negative organisms and antibiotics, such as β‐lactams and glycopeptides, indeed the key drug for melioidosis, ceftazidime, target one or more enzymes required for its biosynthesis 4, 5, 6, 7. Due to the success of targeting parts of the PG biosynthetic pathway 7, we focused on the assessment of d‐alanine‐d‐alanine (d‐Ala‐d‐Ala) ligase as a potential target for the development of new drugs for *B. pseudomallei* infections.

d‐Alanine (d‐Ala) contributes a key structural role in the PG layer as a dipeptide, and in lantibiotics 8, 9, 10. The biosynthesis of d‐Ala‐d‐Ala involves an ATP‐dependent ligase (Ddl, http://www.chem.qmul.ac.uk/iubmb/enzyme/EC6/3/2/4.html) 10, 11. In some bacteria, for example *Escherichia coli*, there exist two proteins (*Ec*DdlA and *Ec*DdlB) 12 with a sequence identity of about 30%. At the time of writing, there are 587 reviewed Ddl sequences in UniProt (http://www.uniprot.org), and 18 bacteria presenting two Ddl proteins with sequence identities from 26% to 58%. A search for DdlA and DdlB sequences, performed using NCBI blast 13, identified only DdlB in *B. pseudomallei* (*Bp*Ddl).

Structures of DdlB from several bacteria have been determined in the apo‐form and ligand‐bound complexes 14, 15. Ddl presents the ATP‐grasp family fold 16, and the catalysed reaction follows a sequential ordered mechanism 17, 18. Known inhibitors of Ddl include d‐cycloserine (DCS) 17, 18, 19, phosphinates 15, 20, 21, diazenocarboxamides 22, hydroxyethylamine 23 and boron derivatives of d‐Ala 24. However, there has been little progress in developing highly potent inhibitors of Ddl with efficient engagement of the cellular target. This might be due to the polar nature of these inhibitors that would require active transport for uptake.

Our objective was to characterise and assess *Bp*Ddl as a potential antibacterial drug target and as part of that assessment to initiate a search for new inhibitor scaffolds. We report an efficient recombinant protein production system, purification and crystallisation protocols, two high‐resolution ligand‐bound *Bp*Ddl structures (AMP and AMP+d‐Ala‐d‐Ala) together with comparisons with orthologues, the optimisation of two enzymatic assays for the functional characterisation of the enzyme and development of high throughput (HTP) assays that were applied to selected compound libraries. We used biolayer interferometry (BLI) and enzymatic assays to assess the outcomes of the HTP library screens and a computational assessment of the *Bp*Ddl active site properties.

## Results and Discussion

### Overall subunit structure

**Figure 1:**
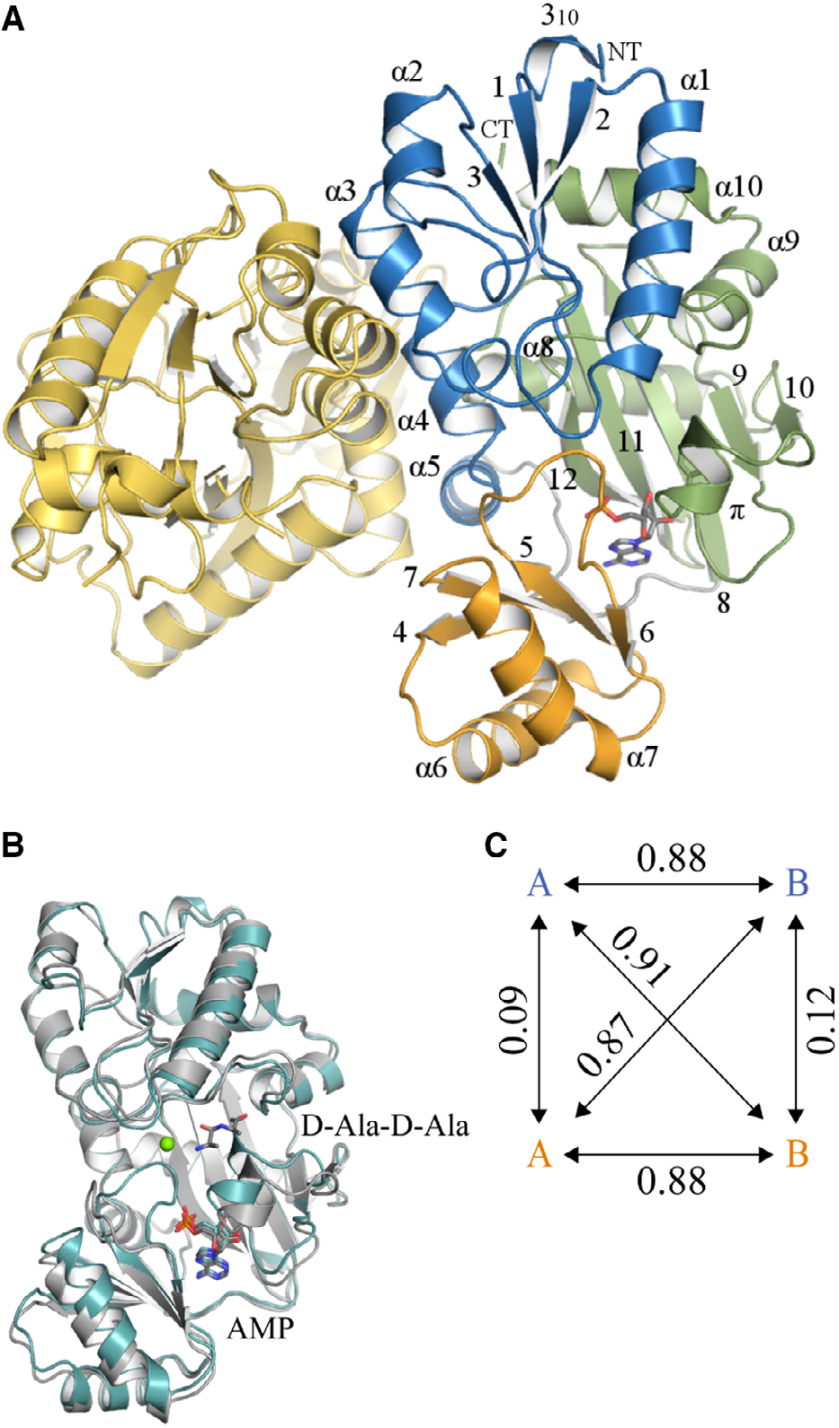
Cartoon representation of the *Bp*Ddl:AMP‐2 structure and structure comparisons. (A) Subunit A is coloured in blue, green and orange, and subunit B in yellow. AMP is represented in sticks. α‐helixes and β‐strands are numbered. 3_10_ and π helixes are marked. The N and C‐termini are labelled as CT and NT. (B) C_α_ overlay of subunit A (in green) and B (grey) from *Bp*Ddl:d‐Ala‐d‐Ala. The green sphere is Mg^2+^. (C) Values for r.m.s.d. (Å) for C_α_ overlay of *Bp*Ddl subunits. *Bp*Ddl:AMP‐2 subunits are coloured in blue and *Bp*Ddl:d‐Ala‐d‐Ala in orange.

Two high‐resolution crystal structures have been determined, *Bp*Ddl:AMP and *Bp*Ddl:AMP:d‐Ala‐d‐Ala, with statistics in Table 1. Ddl is a member of the ATP‐grasp superfamily presents a characteristic and highly conserved fold 16, 25, 26, 27, 28. ATP‐grasp proteins contain three well‐defined domains 16. In the case of Ddl, these are the N‐terminal, central and C‐terminal domains (Fig. 1). *Bp*Ddl secondary structure comprises 10 α‐helixes, one 3_10_‐helix, one π‐helix and 12 β‐strands (Fig. 1A). The N‐terminal segment presents an α/β‐domain whilst the central and C‐terminal domains comprise the ATP‐grasp fold; two α/β‐domains that generate a narrow pocket for the ATP binding. The d‐Ala binding part of the active site is located in the C‐terminal domain. Two loops are implicated in the binding of the co‐factor and the substrate. The P‐loop (residues 149–159) is located in the central domain, between β‐strands 5 and 6, and interacts with the γ‐β phosphates of ATP. The ω‐loop is located in the C‐terminal domain and presents a π‐helix configuration involving residues 198–222 (Fig. 1A). At several positions, gross structural differences are noted when comparing subunits of the two *Bp*Ddl structures (Fig. 1B). The position of the central domains and several helices on the N‐terminal and C‐terminal domains, that line the active site, adopt different positions. The Cα comparison illustrates the notable conservation of these structural changes in both *Bp*Ddl models (Fig. 1C).

**Table 1:** Crystallographic statistics

Structure	*Bp*Ddl:AMP	*Bp*Ddl:AMP:d‐Ala‐d‐Ala
PDB code	http://www.rcsb.org/pdb/search/structidSearch.do?structureId=5NRH	http://www.rcsb.org/pdb/search/structidSearch.do?structureId=5NRI
Space group	*P*2_1_	*P*2_1_
Wavelength (Å)	0.9174	0.9174
Unit cell dimensions	69.65, 61.15, 70.08	69.64, 61.13, 69.67
*a*,* b*,* c* (Å), β (°)	90.17	90.31
Resolution range^a^ (Å)	70.08–1.30	69.97–1.50
No. reflections	672 251	283 077
Unique reflections	141 049	90 609
Completeness (%)	97.6 (95.7)	96.6 (98.3)
*R*_merge_^b^	0.04 (0.66)	0.05 (0.45)
CC_1/2_	0.99 (0.74)	0.99 (0.77)
Redundancy	4.8	3.1
<*I*/σ(*I*)>	15.0 (2.1)	10.5 (2.1)
Wilson *B* (Å^2^)	14.6	15.4
*R*_work_^c^/*R*_free_^d^	0.1459/0.1901	0.1325/0.2077
DPI^e^ (Å)	0.05	0.08
Bond lengths (Å)/angles (°)	0.018/2.247	0.027/2.583
Average *B*‐factors (Å^2^)^f^	23.9	25.2
AMP average *B*‐factors (Å^2^)	13.3	13.7
d‐Ala—d‐Ala average *B*‐factors (Å^2^)	‐	32.4
Protein residues	1412	1345
Water molecules	785	709
Metal ions	1 (Mg^2+^)	1 (Mg^2+^)
Ligands	7 (2 AMP, 2 ${\text{SO}}_{4}^{2-}$, 3 ethylene glycol)	15 (2 AMP, 1 d‐Ala‐d‐Ala, 3 ${\text{SO}}_{4}^{2-}$, 8 ethylene glycol, 1 poly‐ethylene glycol)
Ramachandran analyses		
Favoured regions (%)	98	98
Allowed regions (%)	99.8	99.8

^a^Values in parentheses refer to the highest resolution shell. ^b^ *R*_merge_ = ∑_*hkl*_ *∑*_*i*_ *|*I_*i*_(*hkl*) − <I(*hkl*)>*|/*∑_*hkl*_ *∑*_*i*_ I_*i*_(*hkl*); where I_*i*_(*hkl*) is the intensity of the *i*th measurement of reflection *hkl* and <I(*hkl*)> is the mean value of I_*i*_(*hkl*) for all *i* measurements. ^c^ *R*_*work*_ = ∑_*hkl*_||*F*_o_| − |*F*_c_||/∑|*F*_o_|*,* where *F*_o_ is the observed structure factor and *F*_*c*_ is the calculated structure factor. ^d^ *R*_free_ is the same as *R*_work_ except calculated with a subset, 5%, of data that are excluded from the refinement calculations. ^e^Diffraction Precision Index. ^f^Protein atoms.

### Quaternary structure

**Figure 2:**
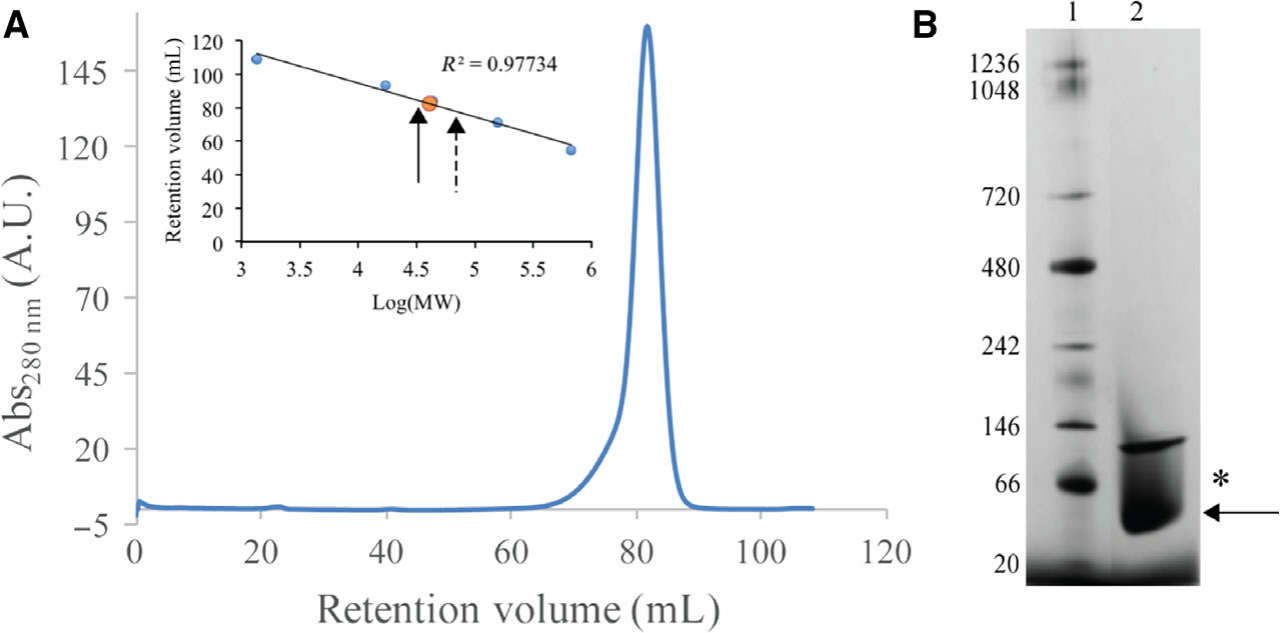
SEC and Native‐PAGE 3‐12% results. (A) SEC chromatogram for *Bp*Ddl. The plot shows the equilibration curve (blue) and *Bp*Ddl peak (orange dot). The expected value for monomers is marked with an arrow and dimer with a dashed arrow. (B) Native‐PAGE. Lane 1: Molecular weight standards. Lane 2: 10 ng of *Bp*Ddl. The arrow indicates the monomer and * the expected value.

In solution, at low concentrations, *Bp*Ddl behaves as a monomer as indicated by SEC. Native‐PAGE on a more concentrated sample indicated monomer as well as the presence of a higher molecular mass species, possibly a dimer (Fig. 2). The crystal structures present a dimer in the asymmetric unit with each subunit aligned perpendicularly to the other (Fig. 1A). The *Bp*Ddl dimer is formed by about 12–13% of the accessible surface area (ASA) of a subunit, which involves residues from α3, α4, α5 and the C‐terminal region of α8. Comparisons with structures from different organisms (Table 2) indicate that the dimer arrangement is shared (data not shown), and the ASA percentage involved in the dimerisation, also called buried surface area (BSA), is similar. These BSA values are lower than expected for a surface with a high probability of forming a multimeric assembly 29, 30. In contrast, the *P*‐value for the probability of having a similar observed solvation free energy gain (Δ^i^G) in other surface areas is lower than 0.5, indicating a higher level of hydrophobicity than expected and it is likely this property that drives oligomerisation 29. *Mycobacterium tuberculosis* Ddl (*Mt*Ddl) 27 has a reduced contact area, with no interactions involving residues on α3 and α8. Also, the *P*‐value is higher for *Mt*Ddl than the other structures. Ddl structures can be grouped according to the additional number of amino acids, compared to *Bp*Ddl, at the C‐terminal end of α10. When extra residues are present, α10 is elongated towards the partner subunit and an increased number of inter‐subunit contacts assist dimer formation. This is noted for *Bacillus anthracis* (*Ba*Ddl), *Streptococcus mutants* (*Sm*Ddl) 31, *Staphylococcus aureus* (*Sa*Ddl) 32, *Coxiella burnetii* (*Cb*Ddl) 27 and *Xanthomonas oryzae* (*Xo*Ddl) 12, where between 8 and 20 additional residues are observed. Table 2 also presents the sequence identity shared with proteins of known structure, which ranges from about 30% to 90%. Despite a wide range of overall sequence identity compared to *Bp*Ddl we note that residues contributing to dimer formation are well conserved, with Gly77, Gly81, Val95, Ala99 and Asp103 present in 12–13 structures of the 14 analysed and three of these residues are also located near the active site binding pockets (Gly77, Val95 and Asp103). Despite differences elsewhere in the subunit–subunit interface, the dimer arrangement is a recurrent feature in Ddl.

**Table 2:** Comparison of *Bp*Ddl with orthologues extracted from the PDB. The first section of the table shows, the percentage of the protein sequence similarity (ID), r.m.s.d. values of the C_α_ overlay of *Bp*Ddl:AMP subunit B and a selected Ddl monomer, and the percentage of residues in the dimer interface (Res_Dimer_) that are conserved and semi‐conserved (in brackets). The second part includes the ASA, BSA values for each subunit (indicated in the subscript), and *P*‐values (probability of the specificity of the interface). BSA is noted as Å^2^ and %. (*) Overlay performed between subunits B from *Bp*Ddl:AMP and *Bp*Ddl:AMP:d‐Ala‐d‐Ala. *B. anthracis* (*Ba*)*, B. ambifaria* (*Bam*), *B. pseudomallei* (*Bp*), *B. xenovorans* (*Bx*), *C. burneii* (*Cb*), *E. coli* (*Ec*), *H. pylori* (*Hp*), *M. tuberculosis* (*Mt*), *S. aureus* (*Sa*), *S. mutants* (*Sm*), *Thermus thermophilus* (*Tt*), *T. caldophilus* (*Tc*), *X. oryzae* (*Xo*) and *Y. pestis* (*Yp*)

Organism	*Bp*	*Bam*	*Bx*	*Yp*	*Ec*	*Tt*	*Tc*
PDB	*Bp*Ddl‐AMP	http://www.rcsb.org/pdb/search/structidSearch.do?structureId=4EG0	http://www.rcsb.org/pdb/search/structidSearch.do?structureId=4EGJ	http://www.rcsb.org/pdb/search/structidSearch.do?structureId=3V4Z	http://www.rcsb.org/pdb/search/structidSearch.do?structureId=4C5C	http://www.rcsb.org/pdb/search/structidSearch.do?structureId=2YZG	http://www.rcsb.org/pdb/search/structidSearch.do?structureId=2FB9
ID (%)	–	92	89	49	48	31	31
r.m.s.d. (Å)	0.12*	0.39	0.63	1.28	1.92	1.84	1.85
Res_Dimer_ (%)	–	100	100	45 (21)	45 (28)	41 (17)	41 (17)
ASA_A_ (Å^2^)	13 896	12 529	12 231	13 489	13 186	14 918	15 331
BSA_A_ (Å^2^)	1793	1287	1089	1331	1666	1372	1368
BSA_A_ (%)	13	10	9	10	13	9	9
*P*‐value_A_	0.007	0.060	0.090	0.022	0.008	0.018	0.044
ASA_B_ (Å^2^)	13 163	12 823	11 431	13 601	13 109	13 983	15 331
BSA_B_ (Å^2^)	1540	1309	1089	1061	1496	1372	1368
BSA_B_ (%)	12	10	10	8	11	10	9
*P*‐value_B_	0.014	0.064	0.094	0.008	0.010	0.018	0.048

Organism	*Xo*	*Ba*	*Sa*	*Cb*	*Sm*	*Mt*	*Hp*
PDB	http://www.rcsb.org/pdb/search/structidSearch.do?structureId=4ME6	http://www.rcsb.org/pdb/search/structidSearch.do?structureId=3R23	http://www.rcsb.org/pdb/search/structidSearch.do?structureId=2I87	http://www.rcsb.org/pdb/search/structidSearch.do?structureId=3TQT	http://www.rcsb.org/pdb/search/structidSearch.do?structureId=3K3P	http://www.rcsb.org/pdb/search/structidSearch.do?structureId=3LWB	http://www.rcsb.org/pdb/search/structidSearch.do?structureId=2PVP
ID (%)	30	34	25	24	28	30	23
r.m.s.d. (Å)	1.54	1.60	1.73	1.95	2.33	1.63	3.76
Res_Dimer_ (%)	38 (21)	24 (28)	24 (28)	24 (24)	24 (21)	17 (14)	17 (34)
ASA_A_ (Å^2^)	16 009	14 940	16 632	16 012	14 731	14 807	16 997
BSA_A_ (Å^2^)	1800	1433	2329	2294	1557	954	1454
BSA_A_ (%)	11	10	14	14	11	6	9
*P*‐value_A_	0.013	0.046	0.176	0.021	0.169	0.382	0.158
ASA_B_ (Å^2^)	16 041	14 842	16 879	15 532	14 669	13 426	16 365
BSA_B_ (Å^2^)	1800	1281	2325	2294	1557	959	1454
BSA_B_ (%)	11	9	14	15	11	7	9
*P*‐value_B_	0.011	0.041	0.083	0.003	0.187	0.333	0.091

### Co‐factor and substrate‐binding pockets

**Figure 3:**
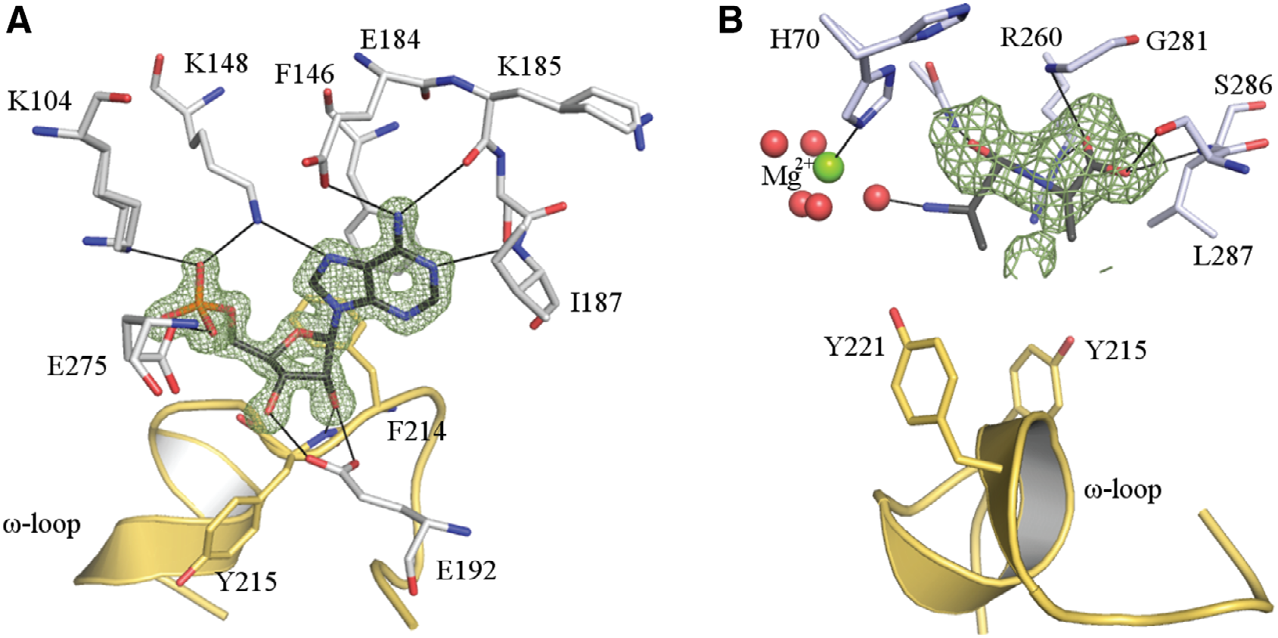
ATP and d‐Ala‐d‐Ala binding pockets. (A) ATP‐binding pocket. AMP is represented as dark grey sticks and the 2Fo‐Fc map (5.0 σ) is shown as a green mesh. (B) d‐Ala binding pocket. The dark grey sticks represent d‐Ala‐d‐Ala. Red circles indicate atoms with zero occupancy. The ω‐loop is represented as a yellow cartoon. The Fo‐Fc map is represented as a green mesh (2.3 σ). Hydrogen bond interactions are indicated with black lines.

Co‐factor and substrate‐binding sites are identified in Figs 1 and 3, the latter also showing electron density associated with the ligands. Whilst the complete AMP is well‐ordered the N, Cα, Cβ atoms of d‐Ala1 are not. The residues that contribute to the formation of these binding sites are highly conserved in Ddl sequences. Indeed, the ATP‐binding site residues (Lys104, Gly153, Glu184, Asp262, Glu275 and Asn277) are also conserved within different functional proteins from the ATP‐grasp superfamily such as acetyl‐CoA carboxylase 16. The recognition of ATP involves residues from the central and C‐terminal domains. These include Lys104, Lys148 and Glu275 binding to the phosphate, and Glu184, Lys185, Ile187, Glu192, Phe214 and Tyr215 that are involved in adenosine recognition. In the case of d‐Ala‐d‐Ala, the key interacting residues are Tyr215, Tyr221, Asp277, Arg260, Gly281, Ser286, Leu287 and also a Mg^2+^‐bound water molecule.

In the substrate‐binding pocket, Lys220, Arg260, Asp277 and Gly281 are strictly conserved and Leu287 in 90% of sequences. These are key residues for substrate binding and catalysis according to the proposed reaction mechanism 17, 18. Conservation is also noted for Phe214, Tyr215 and Tyr221 (40–50% identity) with other aromatic residues. These residues appear to be involved in placing ATP and d‐Ala in the correct position for catalysis. In the ATP‐binding pocket, Lys104, Lys148, Glu184, Glu192 and Glu275 are conserved in between 98% and 100% of the sequences highlighting their importance to co‐factor binding.

**Figure 4:**
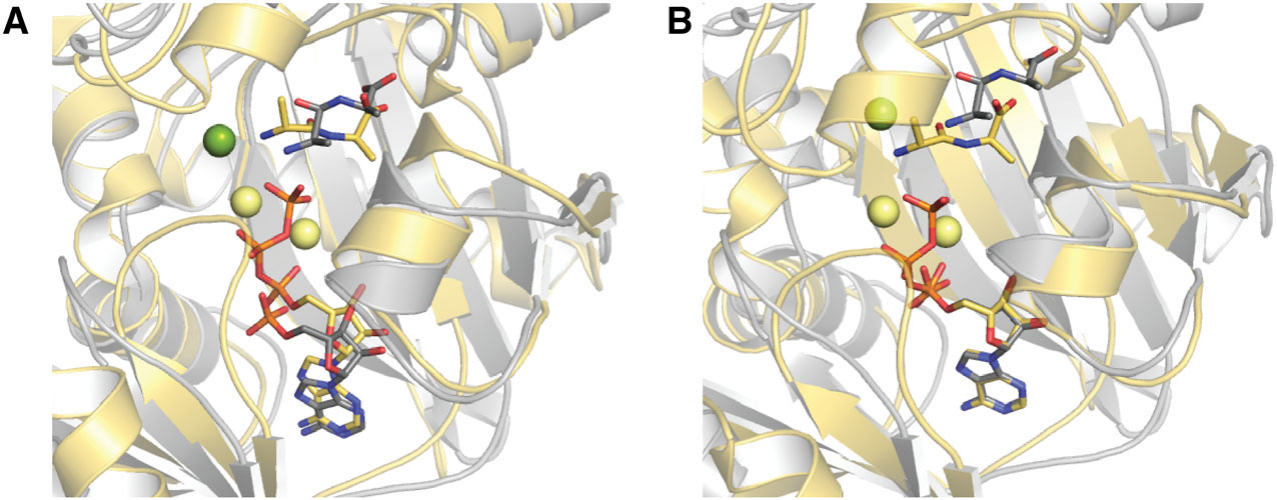
Cα Overlay of *Bp*Ddl:d‐Ala—d‐Ala and *Ec*Ddl (http://www.rcsb.org/pdb/search/structidSearch.do?structureId=4C5C). *Bp*Ddl:d‐Ala—d‐Ala is coloured in grey *Ec*Ddl in yellow. Ligands are represented as sticks. Mg^2+^ are represented as spheres coloured in green (*Bp*Ddl:d‐Ala—d‐Ala) or yellow (*Ec*Ddl).

When *Bp*Ddl:AMP:d‐Ala‐d‐Ala was compared with other product and substrate‐bound structures (from *Ec*Ddl: PDB codes: http://www.rcsb.org/pdb/search/structidSearch.do?structureId=1IOV, http://www.rcsb.org/pdb/search/structidSearch.do?structureId=4C5B, http://www.rcsb.org/pdb/search/structidSearch.do?structureId=4C5C; from *Tt*Ddl: http://www.rcsb.org/pdb/search/structidSearch.do?structureId=2ZDH and http://www.rcsb.org/pdb/search/structidSearch.do?structureId=2ZDQ, including ADP+phosphinate, ADP+d‐Ala, ATP+d‐Ala‐d‐Ala, ADP+d‐Ala, ATP+d‐Ala respectively), differences in the substrate and product binding were found (Fig. 4). The C_α_ overlay of *Bp*Ddl:AMP:d‐Ala‐d‐Ala (subunit A) with these ligand‐bound structures showed the location of d‐Ala‐d‐Ala, though similar, participates in different interactions compared to previously characterised proteins. Additionally, the relative position of the Mg^2+^ is different. In other Ddl structures (e.g. *Ec*Ddl, PDB code http://www.rcsb.org/pdb/search/structidSearch.do?structureId=4C5C), two Mg^2+^ ions interact with Asp262, Glu275 and γ‐β phosphates from ATP. However in *Bp*Ddl:AMP:d‐Ala‐d‐Ala only one Mg^2+^ is observed, interacting with His70. The overall *Bp*Ddl:AMP:d‐Ala‐d‐Ala conformation presents a more open form than in other structures, suggesting it is an intermediate conformation, but it cannot be distinguished if this corresponds to a substrate binding or product release phase.

### Conformational changes

**Figure 5:**
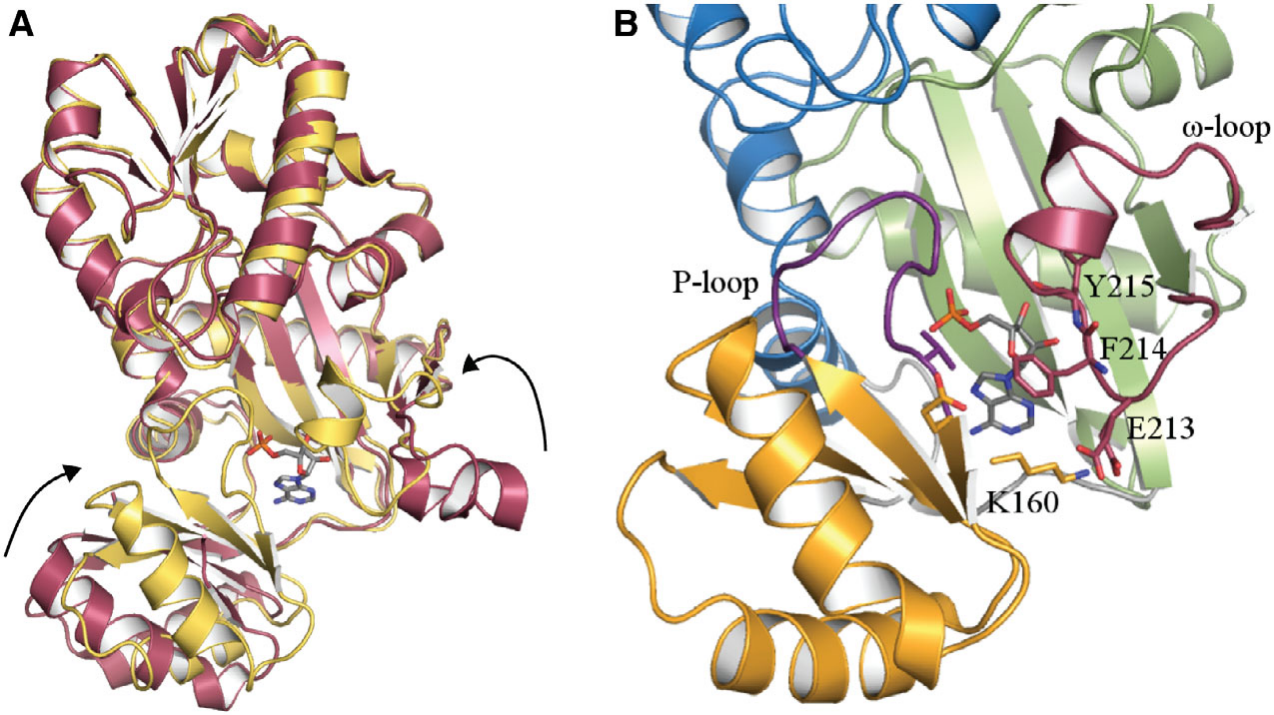
Conformational change. (A) Ribbon diagram of the superposition of the apo‐*Bp*Ddl (http://www.rcsb.org/pdb/search/structidSearch.do?structureId=4EGQ in dark red) and *Bp*Ddl:AMP‐2 (yellow). AMP is represented with sticks. (B) Residues implicated in co‐factor binding. Blue indicates the N‐terminal domain, orange the central domain and green the C‐terminal domain.

Ligand‐induced conformational changes can be observed when comparing the AMP loaded structure with an apo‐form extracted from the PDB (Fig. 5). Binding of AMP generates a more compressed conformation where the central domain moves towards the centre of the structure and the ω‐loop rotates and generates the active site pocket.

ATP binding therefore is likely to stabilise the ω‐loop conformation enforcing interactions with Tyr215 to create a closed conformation. Phe214 is able to interact with a hydrophobic area of the central domain, and Glu213 with Lys160 to assist closing the ATP‐binding pocket. The conformational change in the central domain involves a movement/shift of α7 of about 12.5 and 7.6 Å for α6 (average distances from both subunits) favoured by the AMP and ω‐loop interactions. Additionally, the conformation of the P‐loop is stabilised by AMP interactions, the ω‐loop and the N‐terminal domain residue. In apo‐*Bp*Ddl, the P‐loop is disordered and not included in the model but in the AMP‐bound structures this loop is ordered with an average *B*‐factor of 36.7 Å^2^ (the overall average *B*‐factor value is 23.9 Å^2^). This higher B‐value suggests a greater degree of flexibility in the loop.

There are apo and ligand‐bound structures for *Sa*Ddl (http://www.rcsb.org/pdb/search/structidSearch.do?structureId=2I8C, http://www.rcsb.org/pdb/search/structidSearch.do?structureId=2I80, apo: http://www.rcsb.org/pdb/search/structidSearch.do?structureId=2I87) 32 and *Tt*Ddl ( http://www.rcsb.org/pdb/search/structidSearch.do?structureId=2YZM, http://www.rcsb.org/pdb/search/structidSearch.do?structureId=2YZN, http://www.rcsb.org/pdb/search/structidSearch.do?structureId=2ZDH, http://www.rcsb.org/pdb/search/structidSearch.do?structureId=2ZDG, http://www.rcsb.org/pdb/search/structidSearch.do?structureId=2ZDQ, apo; http://www.rcsb.org/pdb/search/structidSearch.do?structureId=2YZG). In both cases, the conformational changes noted when comparing different states do not appear as pronounced as in *Bp*Ddl. Movement of the *Tt*Ddl central domains involves an α7 translation of 4.7 Å, whereas no differences in *Sa*Ddl are observed. This tends to suggest that the conformational changes are distinct in different organisms. As indicated in ESC library screening in Results and Discussion, purified *Bp*Ddl contained AMP and we were unable to obtain an apo‐*Bp*Ddl crystal form. With this in mind, electron density maps of the apo‐*Bp*Ddl, *Sa*Ddl and *Tt*Ddl structures were inspected to check for missed co‐factors but in all three there was no evidence of anything bound in the ATP pockets.

**Figure 6:**
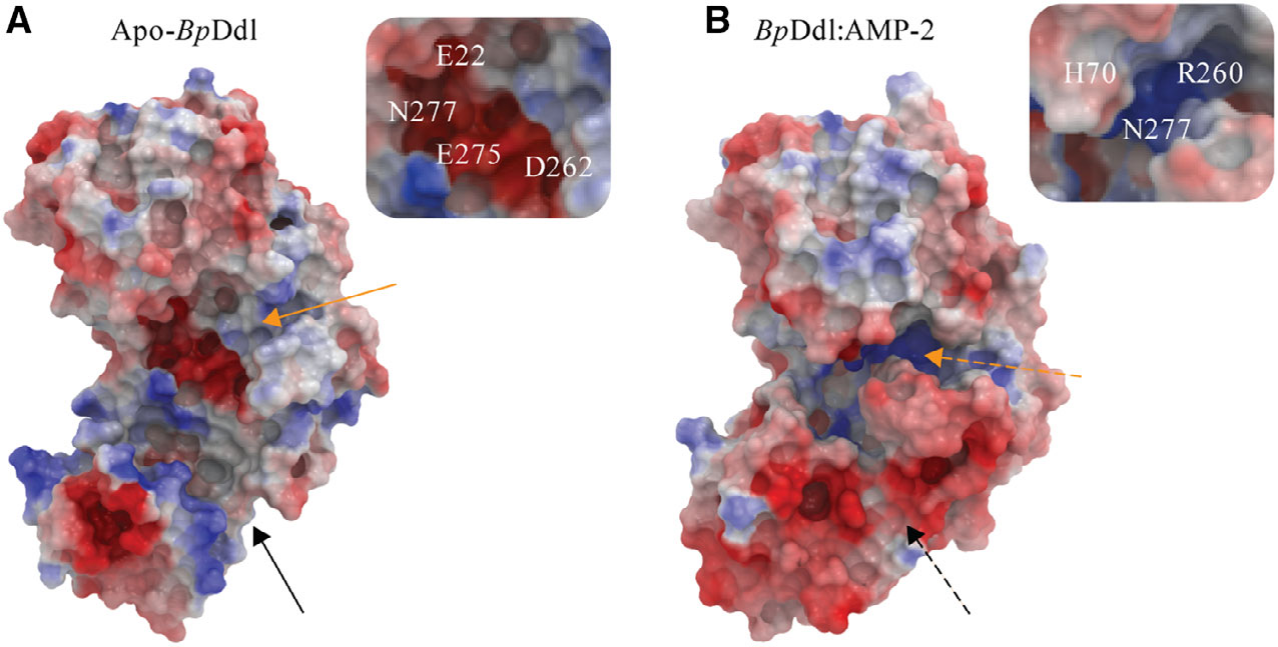
Electrostatic surface. (A) apo‐*Bp*Ddl ( http://www.rcsb.org/pdb/search/structidSearch.do?structureId=4EGQ) and (B) *Bp*Ddl:AMP‐2 structures. Orange arrows indicate the d‐Ala pocket location and black arrows the ATP pocket. Dashed arrows indicate ATP pocket is closed and d‐Ala pocket is formed. On the right side of each surface an enlargement of the d‐Ala pockets are shown and amino acids marked.

A comparison of the electrostatic surfaces in and around the active sites of apo‐*Bp*Ddl and *Bp*Ddl:AMP reveals significant differences (Fig. 6). The d‐Ala pocket is more electropositive in the AMP‐bound structure. Therefore, at pH 7.5, d‐Ala (pI = 6.01) would preferably bind to the *Bp*Ddl:AMP conformation over the apo‐form. This suggests that the d‐Ala pocket is formed after the ω‐loop conformational change. The residues involved in determining the electrostatic properties of the different conformations (Glu22, Asp262, Glu275 and Asn277 in apo‐*Bp*Ddl, and His70, Arg260 and Asn277 in *Bp*Ddl:AMP) are highly conserved. Also, as described previously, His70, Arg260 and Asn277 are key amino acids for Ddl catalysis. This is consistent with the hypothesis that the generation of a favourable environment for catalysis is dependent on ATP binding, in agreement with the sequential ordered mechanism previously characterised 17, 18 where ATP is the first component to bind Ddl.

### Biochemical characterisation

#### Enzyme kinetics

Enzyme activity was investigated using a coupled spectrophotometric assay and a BIOMOL Green assay at pH 7.5 and 8 (Table 3). Using the coupled assay, inhibition of the enzyme activity was observed at high concentrations of ATP and *K*_i,ATP_ was also determined. In the BIOMOL Green assay, high ATP concentrations decrease the signal‐to‐noise ratio due to nonenzymatic hydrolysis and consequently, ATP concentrations did not exceed 1 mm. Nevertheless the *K*_i,ATP_ could not be accurately determined with the BIOMOL assay. The values for *K*_m2_ were consistent between the coupled and the BIOMOL assays. Comparing the *K*_m_ values with other Ddl enzymes, we note more similarity between *Bp*Ddl and *Mt*Ddl or *Ec*Ddl than *Yersinia pestis* Ddl or *Hp*Ddl (Table 1). Indeed, *Hp*Ddl seems to be the least related Ddl, not only displaying a lower sequence identity but also different kinetic parameters.

**Table 3:** Kinetic parameters for Ddl. *K*_m_ values for two d‐Ala and ATP, the *K*_i_ for ATP and the *V*_max_ of the reaction

Detection method	pH	*K*_m1_ (mm)	*K*_m2_ (mm)	*K*_ATP_ (mm)	*K*_i,ATP_ (mm)	*V*max (nmol NAD^+^·min^−1^)
Coupled	8	0.37 ± 0.085	4.19 ± 0.71	0.07 ± 0.01	3.16 ± 0.59	21.85 ± 0.98
Coupled	7.5	0.35 ± 0.03	5.12 ± 1.09	–	–	12.92 ± 1.38
Biomol	8	–	4.9	0.13	–	–
Biomol_TCEP/Tween_	8^a^	–	3.2	0.2	–	–
Biomol_200 μ_ _m__ATP_	8^b^	–	2.3	–	–	–
Biomol_TCEP/Tween_	7.5^a^	–	5.4	0.27	–	–
*Hp*Ddl 26	8	1.89	627^c^	0.000087	–	–
*Ec*Ddl 21	7.8	0.0012	1.13	0.049	–	–
*Mt*Ddl 18	8	0.075 ± 0.01	3.6 ± 0.6	0.31 ± 0.05	–	–
*Yp*Ddl 14	7.8	0.006	0.07	3.2	–	–

^a^In the presence of 1 mm TCEP and 0.01% (w/v) Tween‐20. ^b^ *K*_m2_ in the presence of 200 μm ATP instead of 500 μm ATP. ^c^Maximum d‐Ala concentration tested. (–) Not determined.

We showed that our *Bp*Ddl samples had AMP‐bound, so the *K*_ATP_ determination is compromised. No information about the presence of AMP or similar compounds in *Hp*Ddl, *Ec*Ddl and *Mt*Ddl has been reported. Purification of *Mt*Ddl 17 and *Hp*Ddl 26 was carried out using nickel‐affinity columns whereas *Ec*Ddl used ion exchange 20, 33. It is possible that *Mt*Ddl and *Hp*Ddl also co‐purified with the co‐factor as similar protocols to those we used for *Bp*Ddl were employed; however direct confirmation would have to be sought.

**Figure 7:**
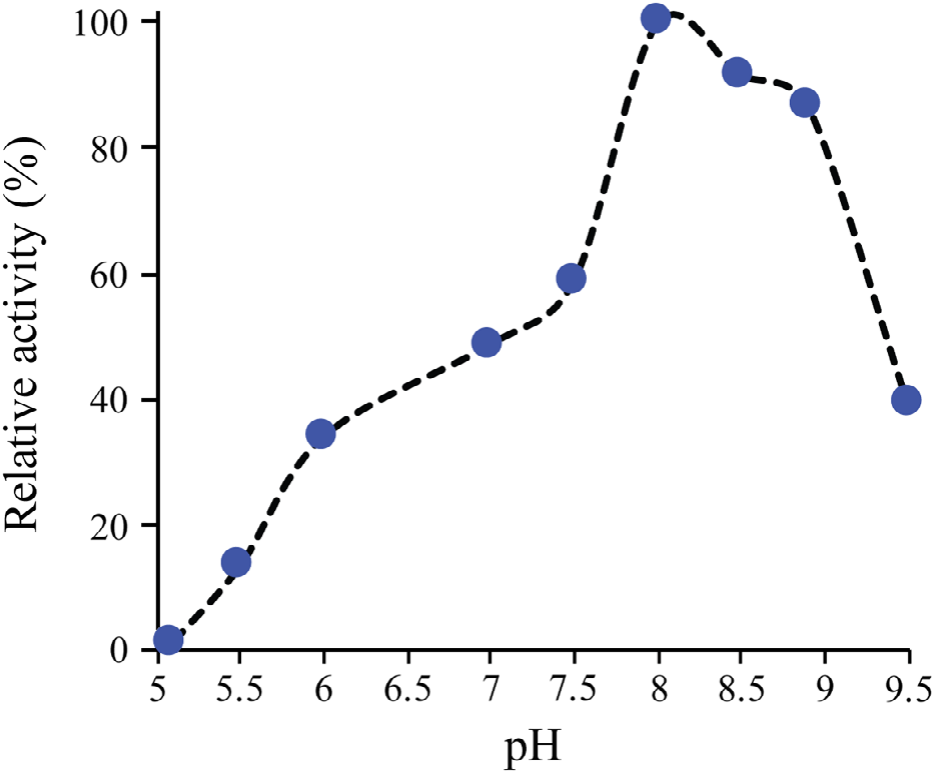
Effect of pH on enzyme activity. The relative activity is set to 100% at maximum level, at pH 8.

The pH optimum for *Bp*Ddl was determined. Differences in the activity due to the change in the pH are observed in the *K*_m2_ and the *V*_max_ determinations. At a lower pH there is an increase of 1.2 times in the *K*_m2_ and 1.7 times decrease in the *V*_max_. To further explore this, activity was measured over a pH range of 5.1–9.5. Regarding this, a test at different pH values has been carried out (Fig. 7). The peak of the maximum activity is observed at pH 8 (100% relative activity) and the activity decreases around 1.7 times at pH 7.5 (56% relative activity). This is consistent with the results from the *K*_m_ experiments.

**Figure 8:**
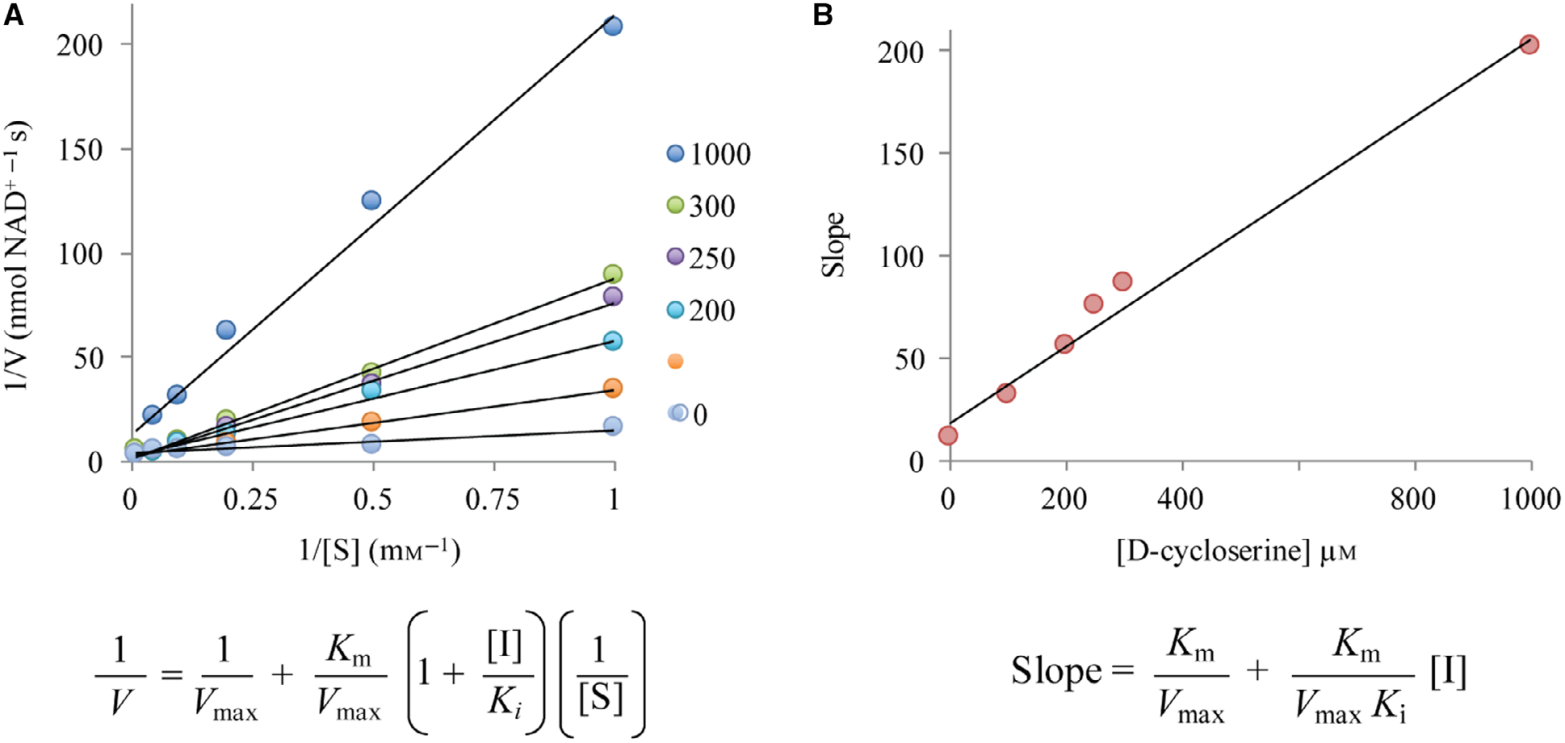
DCS *K*_i_ determination. (A) Double reciprocal plot for the DCS inhibition and the corresponding equation for competitive inhibition. (B) Secondary representation for inhibitory data. Plot of the slopes from the reciprocal plot against the concentration of DCS. From the equation of the slope of this line the *K*_i,DCS_ can be determined as shown in the accompanying equation. *V*,* r*eaction velocity (nmol·min^−1^); *K*_*m*_, Michaelis–Menten constant (M); *V*_max_, maximum velocity of the reaction (nmol·min^−1^); [*S*], substrate concentration (M); [*I*], inhibitor concentration; *K*_i_, inhibitory constant.

Determination of the *K*_i_ and the mechanism of inhibition for DCS were assessed (Fig. 8). DCS appears to be a competitive inhibitor of substrate with a *K*_i,DCS_ value of 100 μm. At the highest DCS concentration tested, 1 mm, a different *V*_max_ was observed what may indicate that the dynamics of binding changes. DCS could be binding both d‐Ala pockets and so display different binding behaviour. For *Mt*Ddl (*K*_i,DSC_ of 25 μm) and *Ec*Ddl (*K*_i,CDS_ of 185 μm), it was reported that slow binding of DCS occurs 19 but not at a high concentration (1000 μm). This DCS concentration dependent behaviour appears to also apply to *Bp*Ddl.

#### Quantification of AMP

Characterisation of structures derived from crystals grown in the presence of different ligands, or none, revealed all had AMP‐bound. This, we presume is derived from the expression host and we decided to quantify the level of AMP in the purified sample. Denatured and folded samples were compared.

**Figure 9:**
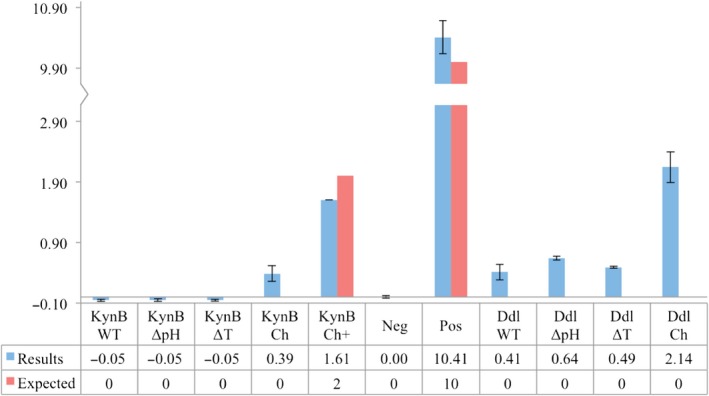
Quantification of AMP. Neg: is the negative control (without enzyme, without AMP). Pos: is the positive control from the standard curve (without enzyme with 10 μm AMP). KynB: is the *Ba*KynB sample. WT: means no pretreatment is performed. ΔT and ΔpH: indicates variations in the temperature or pH were carried out. Ch: indicates digestion with chymotrypsin was used and + is used when 2 μm AMP was added. The error bars correspond to the SD derived from three experiments.

Firstly, controls *Ba*KynB, *Ba*KynB in the presence of 2 μm AMP and *Bp*Ddl itself were denaturated using (a) pH extremes, (b) an elevated temperature and (c) chymotrypsin digestion. The experimental and expected results are shown in Fig. 9. The negative control (in the absence of protein and AMP) and positive control (10 μm AMP in the absence of protein) results were similar to expected values. *Ba*KynB likewise, confirmed there is no AMP‐bound 34. The *Ba*KynB data indicate that the different sample treatments are not interfering with the AMP‐glo assay. The results for *Bp*Ddl demonstrate the sample has been co‐purified with AMP. The presence of AMP without pretreatment of *Bp*Ddl indicates there is some free material in the protein solution (around 0.4 μm) probably due to AMP exchange/release. The sample treated with chymotrypsin presents an AMP concentration of about 2 μm that indicates 1.6 μm (approximately 15 nmol) AMP remains bound to *Bp*Ddl. Taking into account that the concentration of *Bp*Ddl is 25 mg·mL^−1^ (about 20 nmol), then the ratio AMP:*Bp*Ddl suggests almost full occupancy of *Bp*Ddl by the monophosphate. This is consistent with the structural data that show one, well‐ordered AMP per *Bp*Ddl subunit.

**Figure 10:**
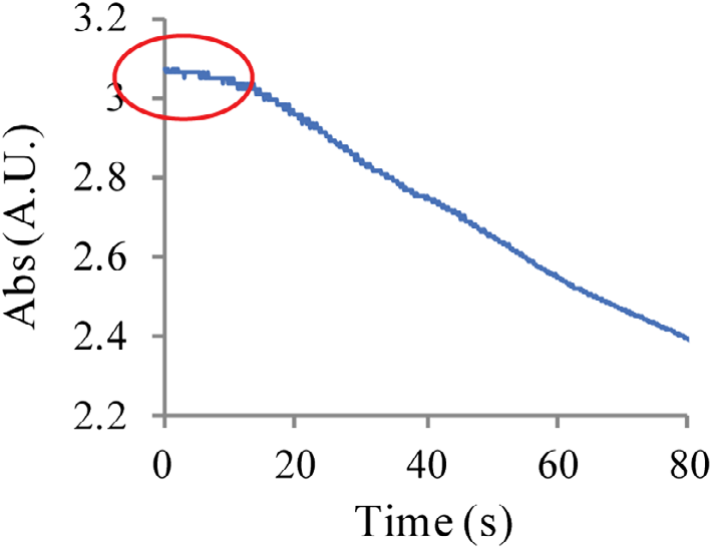
Spectrophotometric assay raw data. A plot of absorbance (340 nm) against time (s). The circle indicates a retarded phase of the reaction.

The presence of AMP in the purified enzyme, presumably acquired from the bacterial expression system has implications for our analyses. The *K*_ATP_ determination and the compound screens therefore have been done mainly with the binary complex *Bp*Ddl:AMP instead of apo‐*Bp*Ddl. Additionally, during the spectrophotometric assay, a retarded phase was observed at the onset of the reaction (Fig. 10). This could be due to diffusion of AMP that has to first occur prior to ATP loading. Also, the fragment screen using BLI was carried out with the closed *Bp*Ddl conformation and that might explain why a relatively low ATP‐binding signal was measured.

An apo‐*Bp*Ddl structure (PDB code http://www.rcsb.org/pdb/search/structidSearch.do?structureId=4EGQ) has been determined by the Seattle Structural Genomics Center for Infectious Disease. Electron and difference density maps in the ATP‐binding sites were inspected to ascertain if a ligand might be present. There were no significant features so we conclude this is a *bone fide* apo‐*Bp*Ddl form. It is unclear why in our structures AMP is present and this may be due to different purification or crystallisation protocols. The PDB entry stipulates that the apo‐form crystal was grown at a basic pH (0.1 m CHES pH 9.5, 1 m Na^+^‐K^+^ tartrate and 0.2 m Li_2_SO_4_) whereas the crystals obtained in our work were obtained at pH 5.5. There are no details of the apo‐form purification. Whilst it is not uncommon for a purified protein to acquire ligands during recombinant protein purification as exemplified by human PPAR‐β/δ binding endogenous fatty acids of *E. coli* 35, it is intriguing in this case. We speculate that this might be indicative of a mechanism whereby the levels of AMP:ATP could be networked to the control exercised by the second messenger cyclic di‐AMP to regulate peptidoglycan biosynthesis 36. The role of AMP and derivatives is coming under increased scrutiny in this aspect 37.

### Compound library screens

We screened three compound libraries. BLI was used in conjunction with a fragment library whilst the malachite green‐based assay was applied to the ESC and small diversity set libraries.

#### BLI: Fragment library screen

**Figure 11:**
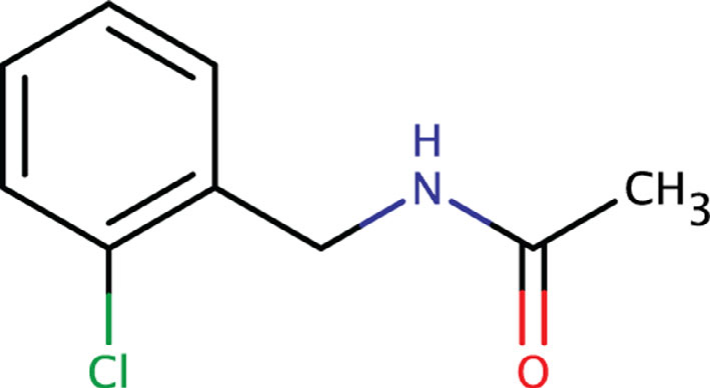
The structure of one compound identified in the fragment screen.

*Bp*Ddl was interrogated with a fragment library of around 700 compounds, using a BLI binding assay. A total of 11 hits were selected for a secondary inhibition assay at 500 μm. A single compound (Fig. 11) gave enzyme inhibition with a *K*_i_ value of 10 mm. Attempts to obtain a structure of the hit in complex with the enzyme by co‐crystallisation failed.

#### ESC library screening

**Figure 12:**
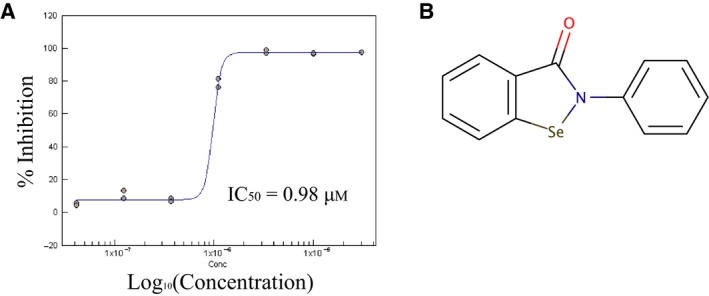
ELF hit SAM001247071 dose–response curve. (A) Dose–response curve of compound SAM001247071. (B) Compound structure.

The optimised HTP assay had a *Z*′ score of 0.8 and a signal‐to‐noise ratio of 5 after 80 min of reaction. Initially, the experiments were run in the absence of TCEP and Tween‐20. To target the substrate and co‐factor binding sites, a concentration of d‐Ala and ATP close to the *K*_m_ values was used. A library of 6775 compounds, comprising well‐known drugs, and, for control purposes, known aggregators, was tested. A total of 74 hits were selected and re‐tested using the BIOMOL Green assay. Results showed no interference by any of the compounds but that 61 appeared to be false positives leaving 13 compounds for further investigation. The dose–response experiment for the hits was performed in the presence of 1 mm TCEP and 0.01% (w/v) Tween‐20 and no inhibition was observed. Thus the redox environment may affect the stability of the compounds and/or induce aggregation. Indeed, inspection of the chemical structures suggested they could also be aggregators 38 and this was further supported by the dose–response profile from one of the compounds (SAM001247071, Fig. 12), which is characteristic of this issue 39. Although our assay appears to be reliable no useful hits were obtained.

#### DDU small diversity set screening

Due to the high similarity of the ATP‐binding pockets between different functional proteins (glutathione synthetase 40, ATP‐citrate lyase 41, succinyl‐CoA ligase 42 and kinases 43), targeting this pocket might result in inhibition of human enzymes with toxic side effects. To avoid this, the inhibitor search was focused on obtaining new inhibitors targeting the d‐Ala binding pocket. The DDU diversity set screen was selected to inform on this part of the active site. A new optimisation of the HTP assay was carried out to establish screening conditions to target this pocket. The reaction was linear for 20 min, the signal‐to‐noise ratio 6 and the *Z*′ score 0.8–0.9. From the 15 667 compounds tested, four hits were selected on the basis of a 30% inhibitory effect. However, the follow‐up dose–response experiment identified them as false positives.

## Experimental procedures

### Recombinant protein production

An oligonucleotide encoding *Bp*Ddl (Unitprot code: http://www.uniprot.org/uniprot/Q63QJ9, protein mass 33 kDa) was purchased from GeneWiz (Takeley, Essex, UK) and inserted into a modified pET15b vector (Novagen, Madison, WI, USA), that adds an N‐terminal hexahistidine tag followed by a tobacco etch virus (TEV) protease cleavage site before the resulting target protein. The restriction enzymes used were NdeI (at the 3′ DNA end) and BamHI (5′ end). The integrity of the construct was confirmed by sequencing. Recombinant protein production was achieved with BL21(DE3) cells, 1 mm IPTG (final concentration) for induction and incubating overnight (O/N) at 20 °C. Purification involved use of metal ion affinity chromatography with a Ni^2+^‐NTA column (GE Healthcare, Chicago, IL, USA) using buffer A (20 mm Tris‐HCl pH 7.4, 200 mm NaCl, 5 mm MgCl_2_) for sample loading and buffer B (20 mm Tris‐HCl pH 7.4, 200 mm NaCl, 5 mm MgCl_2_, 800 mm imidazole) for elution. The hexahistidine tag was cleaved, during dialysis back into buffer A, using 1 mg of TEV protease per 10 mg of *Bp*Ddl, incubated O/N at 4 °C. A second round of affinity chromatography with a Ni^2+^‐NTA column removed the tag and the TEV protease, itself carrying a hexahistidine tag, and fractions carrying the purified protein were pooled. Protein purity and molecular weight were assessed by SDS/PAGE. Protein concentration was determined using the theoretical molar extinction coefficient ε(*Bp*Ddl) = 27 515 m ^−1^·cm^−1^ as calculated in protparam 44. Quaternary structure was investigated using native‐PAGE (Novex, ThermoFisher Scientific, Waltham, MA, USA) and size‐exclusion chromatography (SEC) using buffer A and the HiLoad 16/60 superdex 200 prep grade column (GE Healthcare).

### Crystallisation

The protein sample was concentrated to 25 mg·mL^−1^ in buffer A. Initially, thin needle shaped crystals grew in sitting drops plates at 18 °C after incubating the protein (at different protein : reservoir ratios) with a reservoir containing 0.2 m Li_2_SO_4_, 0.1 m Bis‐Tris pH 5.5 and 25% (w/v) PEG 3350. These crystals were crushed to provide microseeds. Hanging drops were set up using a protein : reservoir ratio of 1 : 1 (in a final volume of 4 μL) plus 0.5 μL of crystal seeds. Crystals grew at 18 °C under various conditions: 0.05–0.3 m Li_2_SO_4_, 0.1 m Bis‐Tris pH 5.5 and 15–30% (w/v) PEG 3350. Co‐crystallisation with different ligand combinations was attempted using *Bp*Ddl incubated for 30 min on ice in the presence of different ligand combinations (e.g. ATP, DCS, d‐Ala+ADP, d‐Ala‐d‐Ala+AMP, DCS+AMP, DCS+AMPPNP). Numerous crystals were analysed but only two structures are reported, a binary complex with AMP and a ternary complex with d‐Ala‐d‐Ala+AMP. In all other cases only AMP was observed in the active site and this will be discussed further below.

### Diffraction, data collection and structural analyses

Optimised crystals, grown in the presence of ATP, were tested in‐house using a M007HF X‐ray generator with a Saturn 944HG+ CCD detector (Rigaku‐Americas, Woodlands, TX, USA) and subsequently used to record a 2.0 Å resolution data set on ESRF beamline BM30‐A. The data were indexed and integrated using xds 45, then scaled and analysed using aimless 46. The Matthews coefficient (*V*_m_ of 2.27 Å^3^·Da^−1^ with bulk solvent of about 50%) suggested two molecules in the asymmetric unit. Two subunits were positioned with phasermr 47 using only the protein components of the 2.2 Å resolution apo‐*Bp*Ddl structure (PDB code http://www.rcsb.org/pdb/search/structidSearch.do?structureId=4EGQ, subunit A). Rigid body refinement followed by cycles of restrained refinement using refmac 48, electron and difference density map inspections, and model manipulations were made with coot 49. AMP, not ATP, was observed in the active site and the end result was a 2.0 Å resolution structure of this binary complex. Other crystals, of putative apo‐enzyme and grown in the presence of different ligands provided 13 data sets on Diamond beamline I04‐1. Data were processed using XDSgui and imolsflm 50, and scaled and merged with aimless. All crystals were isomorphous with the initial structure. These data produced two structures. In one case, that of a crystal obtained in the presence of ADP and d‐Ala, data extending to 1.3 Å resolution were recorded and molecular replacement was performed with the refined subunit A from the binary complex. This structure only showed AMP‐bound in the active site and hence, due to a superior resolution, supersedes the first; this structure is *Bp*Ddl:AMP. A 1.5 Å resolution structure of a ternary complex of *Bp*Ddl:AMP:d‐Ala‐d‐Ala was the second structure. This was refined starting from the *Bp*Ddl:AMP model. Tight, noncrystallographic symmetry (NCS) restraints were employed at the onset of the refinements and these were released once solvents and ligands were included. Side‐chain rotamers, water molecules and ligands, for example, ethylene glycol, were added when the protein models were completed and then anisotropic thermal parameters were included in the model. In the case of the ternary complex, a difference Fourier map suggested the presence of d‐Ala‐d‐Ala. A model with full occupancy for the complete ligand resulted in negative difference density at the N‐terminus of the dipeptide. However, if only the second d‐Ala was refined, positive difference density was observed. We conclude that the first d‐Ala (d‐Ala1) is not fully ordered and although included in the model we assigned zero occupancy. A Mg^2+^ ion is present near His70 (subunit A). Two strong difference density features, in *Bp*Ddl:AMP (near residues 293 from subunit A, 221 from subunit B) were interpreted as ${\text{SO}}_{4}^{2-}$ ions. Three such examples were noted in *Bp*Ddl:AMP: d‐Ala‐d‐Ala (near residues 293 from subunit A, 221 and 290 from subunit B). The sulphates near residue 221 occupy the d‐Ala binding pocket. Refinement was concluded when no improvements resulted for *R*_free_ and *R*_work_ values, when density maps indicated no further changes were justified and that satisfactory model geometry was obtained. For the other data sets, omit maps were inspected after some refinement and AMP was noted in all structures, irrespective of the ligands that had been added or when an apo‐structure was sought. This indicated that AMP might be present in the protein solution, a point we had to investigate (see below).

### Model analyses and informatics

molprobity 51 was used to assess model geometry. Secondary structure was inspected using dssp 30, the dimer interface was analysed using pisa 29, 52 and molsoft icm ( http://www.molsoft.com), and *B*‐factors analysed using Baverage from the ccp4i suite. Figures were prepared using aline 53, pymol (http://www.pymol.org) and molsoft icm. The DALI server 54 was used for comparative purposes whilst electrostatic surfaces and properties of the enzyme active site were determined using molsoft icm.

A comparison of Ddl sequences was carried out. First, a search for relevant sequences in UniProt (http://www.uniprot.org) was conducted. The identified sequences were grouped by 50% identity and aligned using clustal omega 55 with group representatives (based on 588 sequences).

### Enzymatic assays

A coupled spectrophotometric assay was initially optimised for characterising the enzyme. To support the HTP screening of compound libraries, a malachite green‐based assay was developed.

#### A coupled spectrophotometric assay

##### *K*_m_ determination

The activity of *Bp*Ddl was measured using a spectrophotometric assay 17. The assay buffer contained 100 mm Tris‐HCl pH 8, 100 mm KCl, 10 mm MgCl_2_, 2.5 mm phosphoenolpyruvate (PEP), 0.4 mm NADH, 5 μL·mL^−1^ phosphoglycerate kinase/lactate dehydrogenase (PK/LDH) (Merck & Co, Kenilworth, NJ, USA) and 30 μm *Bp*Ddl. Additionally, to determine *K*_m_ for d‐Ala and ATP, 500 μm ATP and variable concentrations of d‐Ala (0.1, 0.3, 0.5, 0.7, 1, 1.5, 2, 2.5, 3, 5, 10, 20 and 100 mm) or 10 mm d‐Ala and variable concentrations of ATP (0.1, 0.2, 0.5, 1.2 and 3 mm) were added respectively. Ten times (10×) concentrated d‐Ala/ATP stocks were prepared for each assay so the volume added was constant. The final reaction volume was 100 μL, and the reaction started by adding the enzyme. All components were incubated for 30 min at 30 °C and then the assay was performed in triplicate. Negative controls included the master mix with *Bp*Ddl and without substrate, and the master mix without *Bp*Ddl and with d‐Ala/ATP. No decrease in absorbance was detected in the negative controls during the assay period. The positive control was the PK/LDH combination and instead of ATP, 0.4 mm ADP was added. Initial velocities were obtained by measuring the steady‐state rate for 60 s and determination of the *K*_m_ values [*K*_m1_(d‐Ala), *K*_m2_(d‐Ala), *K*_m_(ATP)] was carried out as previously described 17. Inhibition of the enzyme activity at high ATP concentrations was observed, and the *K*_i,ATP_ calculation was carried out with graphpad (http://www.graphpad.com).

##### *Bp*Ddl inhibition

Inhibition experiments were performed in the presence of different inhibitor concentrations to determine *K*_m2_(d‐Ala). In the case of DCS the concentrations used were 100, 200, 250, 300, 500 and 1000 μm. Stocks (10×) for each inhibitor concentration were prepared. Each *K*_i_ determination was conducted for about 12 h. To check for time‐dependent loss of *Bp*Ddl activity, an assay without inhibitors was performed before and after the inhibition studies. *K*_i_ calculations were performed using double reciprocal plots 17. To check for inhibition of any of the coupled enzymes, a cross‐validation assay was performed. The assay buffer in this case contained 100 mm Tris‐HCl pH 8, 100 mm KCl, 10 mm MgCl_2_, 2.5 mm PEP, 0.4 mm NADH, 5 μL·mL^−1^ PK/LDH mixture and 0.5 mm ADP. Concentrations of 0.5 and 10 mm DCS (from stock 1 m) and a positive control (without inhibitor) were tested. No inhibition was detected. A potential inhibitor was identified (see below) and the *K*_i_ determination was carried out as for DCS using final concentrations of 200, 500, 2000 and 5000 μm.

##### The pH profile of *Bp*Ddl activity

The reaction mixture contained 100 mm of the selected buffer, 10 mm KCl, 10 mm MgCl_2_, 2.5 mm PEP, 0.4 mm NADH, 5 μL·mL^−1^ PK/LDH mix, 0.5 mm ATP, 10 mm d‐Ala and 20 μm *Bp*Ddl (added to start the reaction). The buffers used are: Bis‐tris pH 5.5, Tricine pH 5.1, MES pH 6.0, HEPES pH 7.5, Tris‐HCl pH 7.5, Tris‐HCl pH 8.0, Tris‐HCl pH 8.5, HEPES pH 8.5, BICINE pH 9.0 and CHES pH 9.5. The maximum reaction rate was used to define 100% activity.

#### Malachite green‐based assay

##### *K*_*m*_ determination

The release of phosphate after ATP hydrolysis can be measured using malachite green (BIOMOL green, ENZO life sciences, Farmingdale, NY, USA). This set of experiments and a compound library screen (see ESC library screen using the malachite green‐based assay in Experimental procedures) were carried out at the European Screening Centre (ESC, Newhouse, UK). The assays were run at room temperature in a final volume of 30 μL, in clear bottom 384 well plates (Greiner, Kremsmunster, Austria). The assay buffer to determine the *K*_m2_(d‐Ala) and *K*_m_(ATP) contained 100 mm Tris‐HCl pH 8, 10 mm KCl, 10 mm MgCl_2_, 0.4 nm *Bp*Ddl, variable concentrations of d‐Ala (serial 1/4 dilutions from top concentration of 10 mm) or ATP (serial 1/4 dilutions from top concentration of 0.5 mm) and 0.5 mm ATP or 10 mm d‐Ala respectively. The time points used in these experiments were as follows: 10 s, 30 s, 1, 1.5, 2, 3, 5, 7, 10, 15 and 20 min. To avoid nonspecific ATP hydrolysis due to the low pH of the BIOMOL green reagents, tri‐sodium citrate was added to each reaction at a final concentration of 0.65% (w/v). Each experiment included four replicates. Absorbance was recorded at 620 nm using a PerkinElmer EnVision plate reader (PerkinElmer, Waltham, MA, USA). For the calculations of d‐Ala *K*_m_, only *K*_m2_ was taken into account and data were analysed with the Michaelis–Menten equation. High ATP concentrations are incompatible with the BIOMOL Green assay hence enzyme inhibition by this compound was not a factor and *K*_ATP_ was determined using the Michaelis–Menten equation. Regarding the *K*_m2_(d‐Ala) and *K*_m_(ATP) results, the chosen HTP assay conditions were as follows: 100 mm Tris‐HCl pH 8, 10 mm KCl, 10 mm MgCl_2_, 0.4 nm *Bp*Ddl, 200 μm ATP and 5 mm d‐Ala.

To determine whether the assay was linear under the chosen assay conditions, a time course experiment was carried out. A total of 24 reactions were started at time points 2.5 min apart during 90 min, and eight replicates included. Additionally, the same experiment was done in the absence of enzyme. These results were used to determine the *Z*′ score (Eqn (1)) and the signal‐to‐noise ratio of the experiment.

(1) $$Z^{\prime }=1-\frac{3({\text{SD}}_{\max}+{\text{SD}}_{\min})}{\text{mean (max)}-\text{mean (min)}},$$

SD = standard deviation, max = maximum signal, min = minimum signal.

##### Determination of DMSO tolerance and d‐cycloserine IC_50_

Using the HTP assay conditions, a dose–response experiment was performed using variable concentrations of DMSO (from 0% to 27%) or DCS (0–300 μm). A 2% inhibition of enzyme activity was noted at 6% DMSO.

### Compound library screens

#### Fragment library screen using BLI

A fragment library from the Drug Discovery Unit (DDU, University of Dundee) was screened using an OctetRED384 (ForteBio‐Molecular Devices, Fremont, CA, USA). The protein was immobilised on hexahistidine tag high affinity sensors (ForteBio), and the experiment carried out in black plates (Greiner). We used TEV protease as a control and to identify promiscuous binders, and ATP as the control ligand. During the assay performance, we noticed a low binding signal (lower than 0.1 nm) in the presence of ATP. Selected hits were tested for enzyme inhibition using the coupled enzymatic assay at a fixed concentration of 500 μm, in the presence of 2 mm d‐Ala. One compound with an inhibition effect greater than 20% was followed up for *K*_i_ determination.

#### ESC library screen using the malachite green‐based assay

A small selection of compounds from the BioAscent compound cloud and the NCC library of drug compounds from the NIH were tested at a concentration of 10 μm. Each screening plate included positive (normal reaction without compounds added) and negative controls (without enzyme). Compounds were dispensed using a Labcyte Echo acoustic dispenser. The assay was carried out using the HTP conditions and allowed to proceed for 1 h before stopping the reaction by adding BIOMOL green. Data were analysed using ActivityBase XE (http://www.idbs.com). A compound was considered a hit when the normalised percentage effect was higher than the median plus three times the standard deviation. The final DMSO concentration during screening reached 0.075% so the assay has not been compromised. Selected hits were re‐tested using the HTP assay conditions in the presence and absence of enzyme with a final concentration of 20 μm to filter false positives. Compounds inhibiting more than 30% of *Bp*Ddl activity were selected for IC_50_ determination. The dose–response experiment was carried out using the same conditions and in the presence or absence of 0.01% (w/v) Tween‐20 and 1 mm TCEP. Data were analysed using ActivityBase XE and Microsoft EXCEL.

#### Small diversity set screen using the malachite green‐based assay

A small diversity set library was screened under HTP assay conditions modified to target the d‐Ala pocket, at pH 7.5, and including 0.01% (w/v) Tween‐20 and 1 mm TCEP in the assay buffer. Consequently, further assay optimisation was required. *K*_m2_(d‐Ala) and *K*_m_(ATP) at pH 7.5 were determined as described before using 1 nm *Bp*Ddl. The time course experiment was performed with 3, 2 and 1 mm d‐Ala and 500 μm ATP. The compound set was used at a final concentration of 30 μm. The reaction was carried out with 1 mm d‐Ala and 0.5 mm ATP, and incubated for 12 min then absorbance was measured using a PHERAstar (BMG Labtech, Ortenberg, Germany). Data were analysed using ActivityBase XE and Microsoft EXCEL and any compound providing 30% inhibition was considered a hit. Validation of hits was performed by dose–response experiments using different concentrations (1/10 dilutions starting at 100 μm) in duplicate.

### AMP detection

The crystallographic analyses revealed the presence of AMP‐bound to the protein even when no nucleotide was added to the sample. Therefore, the AMP‐Glo kit from Promega (Madison, WI, USA) was used to quantify the presence of AMP in the purified *Bp*Ddl. The reaction was performed in a 96‐well white plate (Greiner), using 25 μL of sample, in triplicate. The signal was measured with a FLUOstar OPTIMA microplate reader (BMG Labtech). The control protein, selected because it does not bind AMP/ADP/ATP, was an amidase called *Ba*KynB 34. Samples were denaturated before AMP quantification using three different treatments. These included heating (20 min at 100 °C prior to the assay performance), exposure to extreme pH (2 min incubation at pH 2, then pH 10, then 7) and digestion with chymotrypsin. The samples used were *Bp*Ddl, *Ba*KynB and *Ba*KynB in the presence of 2 μm AMP (*Ba*KynB‐AMP) as a control. Chymotrypsin was added to a final ratio chymotrypsin : sample of 1 : 20 and the mixtures incubated O/N at 25 °C. Then, the samples were filtered using an Amicon ULTRA 3K concentrator (Merck & Co). The protein concentration was 4 mg·mL^−1^ for *Ba*KynB and 25 mg·mL^−1^ for *Bp*Ddl. Data are presented in Fig. 9.

## Conclusions

We set out to assess, and potentially validate, *Bp*Ddl as a target for the development of the new antibacterial drugs for *B. pseudomallei*. Two high‐resolution crystal structures of *Bp*Ddl in complex with AMP and AMP+ d‐Ala‐d‐Ala were obtained. Comparative studies indicate aspects of Ddl that are well conserved allow us to describe conformational differences that result from ligand binding. The *Bp*Ddl structures provided templates for a computational druggability assessment, but also identified that the enzyme was retaining AMP during isolation. This was confirmed using an AMP detection assay. The co‐purification with AMP suggests that we have assayed a binary complex not the apo‐enzyme. This had not been reported previously and might also apply in other Ddl studies. The *Bp*Ddl activity was measured using two different enzyme assays and showed similar kinetic parameters to two other Ddl enzymes but different from a third. We noted a retarded phase during assay that was probably due to the presence of AMP which first has to vacate the co‐factor binding site prior to catalysis.

High‐throughput binding and enzymatic assays were developed to conduct compound library screens. Despite screening more than 22 500 compounds, we did not identify any good starting points for further study. Whilst our study was underway, AstraZeneca reported a larger compound library screen for *Streptococcus mutans* Ddl but no lead compounds were found 56, which is consistent with our results.

Our overall assessment is that *Bp*Ddl represents a challenging target for drug discovery. However, our progress in enabling ligand‐binding studies of the enzyme means that in future a structure‐based rational approach 57 combining crystallography with computational docking and modelling calculations would be feasible. In similar fashion, further HTP screens can be prosecuted but the composition of the library would have to be carefully considered.

## Conflict of interest

The authors declare no conflict of interest.

## Author contributions

Participated in research design, performed the data analysis and contributed to the writing of the manuscript: LD‐S, LST, SPM, DG, WNH; Conducted experiments: LD‐S, LST, SPM.

## References

[1] Fair RJ & Tor Y (2014) Antibiotics and bacterial resistance in the 21st century. Perspect Medicin Chem 6, 25–64. 25232278 10.4137/PMC.S14459 PMC4159373

[2] Amornchai VWP , Saiprom N , Chantratita N , Chierakul W , Gavin CKW , Koh GCKW , Chaowagul W , Day NPJ , Limmathurotsakul D & Peacock SJ (2011) Survey of antimicrobial resistance in clinical *Burkholderia pseudomallei* isolates over two decades in northeast Thailand. Antimicrob Agents Chemother 55, 5388–5391. 21876049 10.1128/AAC.05517-11 PMC3195054

[3] Schweizer HP (2012) Mechanisms of antibiotic resistance in *Burkholderia pseudomallei*: implications for treatment of melioidosis. Future Microbiol 7, 1389–1399. 23231488 10.2217/fmb.12.116 PMC3568953

[4] Gautam A , Vyas R & Tewari R (2011) Peptidoglycan biosynthesis machinery: a rich source of drug targets. Crit Rev Biotechnol 31, 295–336. 21091161 10.3109/07388551.2010.525498

[5] Moraes GL , Gomes GC , Monteiro de Sousa PR , Alves CN , Govender T , Kruger HG , Maguire GE , Lamichhane G & Lameira J (2015) Structural and functional features of enzymes of *Mycobacterium tuberculosis* peptidoglycan biosynthesis as targets for drug development. Tuberculosis 95, 95–111. 25701501 10.1016/j.tube.2015.01.006 PMC4659487

[6] Liu Y & Breukink E (2016) The membrane steps of bacterial cell wall synthesis as antibiotic targets. Antibiotics (Basel) 5, 28. 10.3390/antibiotics5030028 PMC5039524 27571111

[7] Kohanski MA , Dwyer DJ & Collins JJ (2010) How antibiotics kill bacteria: from targets to networks. Nat Rev Microbiol 8, 423–435. 20440275 10.1038/nrmicro2333 PMC2896384

[8] Cava F , Lam H , de Pedro MA & Waldor MK (2010) Emerging knowledge of regulatory roles of D‐amino acids in bacteria. Cell Mol Life Sci 68, 817–831. 21161322 10.1007/s00018-010-0571-8 PMC3037491

[9] Neuhaus FC & Baddiley J (2003) A continuum of anionic charge: structures and functions of D‐alanyl‐teichoic acids in Gram‐positive bacteria. Microbiol Mol Biol Rev 67, 686–723. 14665680 10.1128/MMBR.67.4.686-723.2003 PMC309049

[10] Bugg TDH , Braddick D , Dowson CG & Roper DI (2011) Bacterial cell wall assembly: still an attractive antibacterial target. Trends Biotechnol 29, 167–173. 21232809 10.1016/j.tibtech.2010.12.006

[11] Bouhss A , Trunkfield AE , Bugg TD & Mengin‐Lecreulx D (2008) The biosynthesis of peptidoglycan lipid‐linked intermediates. FEMS Microbiol Rev 32, 208–233. 18081839 10.1111/j.1574-6976.2007.00089.x

[12] Zawadzke LE , Bugg TD & Walsh CT (1991) Existence of two D‐alanine: D‐ alanine ligases in *Escherichia coli*: cloning and sequencing of the ddlA gene and purification and characterization of the DdlA and DdlB enzymes. Biochemistry 30, 1673–1682. 1993184 10.1021/bi00220a033

[13] Johnson M , Zaretskaya I , Raytselis Y , Merezhuk Y , McGinnis S & Madden TL (2008) NCBI BLAST: a better web interface. Nucleic Acids Res 36, 5–9. 10.1093/nar/gkn201 PMC2447716 18440982

[14] Tran HT , Hong MK , Ngo HP , Huynh KH , Ahn YJ , Wang Z & Kang LW (2016) Structure of D‐alanine‐ D‐alanine ligase from *Yersinia pestis*: nucleotide phosphate recognition by the serine loop. Acta Crystallogr D 72, 12–21. 10.1107/S2059798315021671 26894530

[15] Fan C , Park II‐S , Walsh CT & Knox JR (1997) D‐alanine:D‐alanine ligase: phosphonate and phosphinate intermediates with wild type and the Y216F mutant. Biochemistry 36, 2531–2538. 9054558 10.1021/bi962431t

[16] Fawaz MV , Topper ME & Firestine SM (2011) The ATP‐grasp enzymes. Bioorg Chem 39, 189–191. 10.1016/j.bioorg.2011.08.004 PMC3243065 21920581

[17] Prosser GA & de Carvalho LPS (2013) Kinetic mechanism and inhibition of *Mycobacterium tuberculosis* D‐alanine:D‐alanine ligase by the antibiotic D‐ cycloserine. FEBS J 280, 1150–1166. 23286234 10.1111/febs.12108

[18] Prosser GA & de Carvalho LPS (2013) Reinterpreting the mechanism of inhibition of *Mycobacterium tuberculosis* D‐alanine:D‐alanine ligase by D‐ cycloserine. Biochemistry 52, 7145–7149. 24033232 10.1021/bi400839f PMC3944805

[19] Batson S , de Chiara C , Majce V , Lloyd AJ , Gobec S , Rea D , Fülöp V , Thoroughgood CW , Simmons KJ , Dowson CG *et al* (2017) Inhibition of D‐Ala:D‐Ala ligase through a phosphorylated form of the antibiotic D‐cycloserine. Nat Commun 8, 1939. 29208891 10.1038/s41467-017-02118-7 PMC5717164

[20] Parsons WH , Patchett WH , Bull HG , Schoen WR , Taub D , Davidson J , Combs PL , Springer JP , Gadebusch H , Wiessberger MEV *et al* (1988) Phosphinic acid inhibitors of D‐alanyl‐ D‐alanine ligase. J Med Chem 31, 1772–1778. 3137344 10.1021/jm00117a017

[21] Ellsworth BA , Tom NJ & Bartlett PA (1996) Synthesis and evaluation of inhibitors of bacterial D‐alanine:D‐alanine ligases. Chem Biol 3, 37–44. 8807826 10.1016/s1074-5521(96)90082-4

[22] Kovač A , Majce V , Lenarsic R , Bombek S , Bostock JM , Chopra I , Polanc S & Gobec S (2007) Diazenedicarboxamides as inhibitors of D‐alanyl‐D‐lactate ligase. Bioorg Med Chem Lett 17, 2047–2054. 17267218 10.1016/j.bmcl.2007.01.015

[23] Sova M , Cadez G , Turk S , Majce V , Polanc S , Batson S , Lloyd AJ , Roper DI , Fishwick CW & Gobec S (2009) Design and synthesis of new hydroxyethylamines as inhibitors of D‐alanyl‐D‐lactate ligase (VanA) and D‐ alanyl‐D‐alanine ligase (DdlB). Bioorg Med Chem Lett 19, 1376–1379. 19196510 10.1016/j.bmcl.2009.01.034

[24] Putty S , Rau A , Jamindar D , Pagano P , Quinn TM , Schewiezer HP & Gutheil WG (2011) Characterization of D‐boroAla as a novel broad‐spectrum antibacterial agent targeting D‐Ala‐D‐Ala ligase. Chem Biol Drug Des 78, 757–763. 21827632 10.1111/j.1747-0285.2011.01210.x PMC3193593

[25] Lee JH , Na Y , Song HE , Kim D , Park BH , Rho SH , Im YJ , Kim MK , Kang GB , Lee DS *et al* (2006) Crystal structure of the apo form of D‐alanine:D‐alanine ligase (Ddl) from *Thermus caldophilus*: a basis for the substrate‐induced conformational changes. Proteins 64, 1078–1082. 16779845 10.1002/prot.20927

[26] Wu D , Zhang L , Kong Y , Du J , Chen S , Chen J , Ding J , Jiang H & Shen H (2008) Enzymatic characterization and crystal structure analysis of the D‐alanine‐D‐alanine ligase from *Helicobacter pylori* . Proteins 72, 1148–1160. 18320587 10.1002/prot.22009

[27] Bruning JB , Murillo AC , Chacon O , Barletta RG & Sacchettini JC (2011) Structure of the *Mycobacterium tuberculosis* D‐alanine:D‐alanine ligase, a target of the antituberculosis drug D‐cycloserine. Antimicrob Agents Chemother 55, 291–301. 20956591 10.1128/AAC.00558-10 PMC3019625

[28] Doan TT , Kim JK , Ngo HP , Tran HT , Cha SS , Min Chung K , Huynh KH , Ahn YJ & Kang LW (2014) Crystal structures of D‐alanine‐D‐alanine ligase from *Xanthomonas oryzae pv. oryzae* alone and in complex with nucleotides. Arch Biochem Biophys 545, 92–99. 24440607 10.1016/j.abb.2014.01.009

[29] Krissinel E & Henrick K (2005) Detection of protein assemblies in crystals In Computational Life Sciences: Lecture Notes in Computer Science. 3695 (Berthold MR, Glen RC, Diederichs K, Kohlbacher O & Fischer I, eds), pp. 163–174. Springer, Berlin.

[30] Touw WG , Baakman C , Black J , te Beek TAH , Krieger E , Joosten RP & Vriend G (2015) A series of PDB‐related databanks for everyday needs. Nucleic Acids Res 43, 364–368. 10.1093/nar/gku1028 PMC4383885 25352545

[31] Lu Y , Xu H & Zhao X (2010) Crystal structure of the apo form of D‐alanine:D‐alanine ligase (Ddl) from *Streptococcus mutants* . Protein Pept Lett 17, 1053–1057. 20522004 10.2174/092986610791498858

[32] Liu S , Chang JS , Herberg JT , Homg M‐M , Tomich PK , Lin AH & Marotti KR (2006) Allosteric inhibition of *Staphylococcus aureus* D‐alanine:D‐alanine ligase revealed by crystallographic studies. Proc Natl Acad Sci USA 103, 15178–15183. 17015835 10.1073/pnas.0604905103 PMC1622796

[33] Franklin MC , Cheung J , Rudolph MJ , Burshteyn F , Cassidy M , Gary E , Hillerich B , Yao ZK , Carlier PR , Totrov M *et al* (2015) Structural genomics for drug design against the pathogen *Coxiella burnetii* . Proteins 83, 2124–2136. 26033498 10.1002/prot.24841

[34] Díaz‐Sáez L , Srikannathasan V , Zoltner M & Hunter WN (2014) Structures of bacterial kynurenine formamidase reveal a crowded binuclear zinc catalytic site primed to generate a potent nucleophile. Biochem J 462, 581–589. 24942958 10.1042/BJ20140511 PMC4243253

[35] Fyffe SA , Alphey MS , Buetow L , Smith TK , Ferguson MA , Sørensen MD , Björkling F & Hunter WN (2006) Recombinant human PPAR‐beta/delta ligand‐binding domain is locked in an activated conformation by endogenous fatty acids. J Mol Biol 356, 1005–1013. 16405912 10.1016/j.jmb.2005.12.047

[36] Barreteau H , Kovac A , Boniface A , Sova M , Gobec S & Blanot D (2008) Cytoplasmic steps of peptidoglycan biosynthesis. FEMS Microbiol Rev 32, 68–207. 10.1111/j.1574-6976.2008.00104.x 18266853

[37] Whiteley AT , Garelis NE , Peterson BN , Choi PH , Tong L , Woodward JJ & Portnoy DA (2017) c‐di‐AMP modulates *Listeria monocytogenes* central metabolism to regulate growth, antibiotic resistance and osmoregulation. Mol Microbiol 104, 212–233. 28097715 10.1111/mmi.13622 PMC5391996

[38] Baell JB & Holloway GA (2010) New substructure filters for removal of pan assay interference compounds (PAINS) from screening libraries and for their exclusion in bioassays. J Med Chem 53, 2719–2740. 20131845 10.1021/jm901137j

[39] Shoichet BK (2006) Interpreting steep dose‐response curves in early inhibitor discovery. J Med Chem 49, 7274–7277. 17149857 10.1021/jm061103g

[40] Dinescu A , Cundari TR , Bhansali VS , Luo JL & Anderson ME (2004) Function of conserved residues of human glutathione synthetase: implications for the ATP‐grasp enzymes. J Biol Chem 279, 22412–22421. 14990577 10.1074/jbc.M401334200

[41] Sun T , Hayakawa K & Fraser ME (2011) ADP‐Mg^2+^ bound to the ATP‐grasp domain of ATP‐citrate lyase. Acta Crystallogr F 67, 1168–1172. 10.1107/S1744309111028363 PMC3212355 22102020

[42] Jaberi E , Chitsazian F , Ali Shahidi G , Rohani M , Sina F , Safari I , Malakouti Nejad M , Houshmand M , Klotzle B & Elahi E (2013) The novel mutation p.Asp251Asn in the β‐subunit of succinate‐CoA ligase causes encephalomyopathy and elevated succinylcarnitine. J Hum Genet 58, 526–530. 23759946 10.1038/jhg.2013.45

[43] Josefsen L , Bohn L , Sørensen MB & Rasmussen SK (2007) Characterization of a multifunctional inositol phosphate kinase from rice and barley belonging to the ATP‐grasp superfamily. Gene 397, 114–125. 17531407 10.1016/j.gene.2007.04.018

[44] Gasteiger E , Gattiker A , Hoogland C , Ivanyi I , Appel RD & Bairoch A (2003) ExPASy: the proteomics server for in‐depth protein knowledge and analysis. Nucleic Acids Res 31, 3784–3788. 12824418 10.1093/nar/gkg563 PMC168970

[45] Kabsch W (2010) XDS. Acta Crystallogr D 66, 125–132. 20124692 10.1107/S0907444909047337 PMC2815665

[46] Evans PR (2011) An introduction to data reduction: space‐group determination, scaling and intensity statistics. Acta Crystallogr D 67, 282–292. 21460446 10.1107/S090744491003982X PMC3069743

[47] McCoy AJ , Grosse‐Kunstleve RW , Adams PD , Winn MD , Storoni LC & Read RJ (2007) Phaser crystallographic software. J Appl Crystallogr 40, 658–674. 19461840 10.1107/S0021889807021206 PMC2483472

[48] Murshudov GN , Skubak P , Lebedev AA , Pannu NS , Steiner RA , Nicholls NA , Winn MD , Long F & Vagin AA (2011) REFMAC5 for the refinement of macromolecular crystal structures. Acta Crystallogr D 67, 355–367. 21460454 10.1107/S0907444911001314 PMC3069751

[49] Emsley P , Lohkamp B , Scott WG & Cowtan K (2010) Features and development of Coot. Acta Crystallogr D 66, 486–501. 20383002 10.1107/S0907444910007493 PMC2852313

[50] Leslie AGW & Powell HR (2007) Evolving Methods for Macromolecular Crystallography. 245, pp. 41–51. Springer, Berlin.

[51] Chen VB , Arendall WB , Headd JJ , Keedy DA , Immormino GJK , Murray LW , Richardson JS & Richardson DC (2010) MolProbity: all‐atom structure validation for macromolecular crystallography. Acta Crystallogr D 66, 12–21. 20057044 10.1107/S0907444909042073 PMC2803126

[52] Krissinel E & Henrick K (2007) Inference of macromolecular assemblies from crystalline state. J Mol Biol 372, 774–797. 17681537 10.1016/j.jmb.2007.05.022

[53] Bond CS & Schüttelkopf AW (2009) ALINE: a WYSIWYG protein‐sequence alignment editor for publication‐quality alignments. Acta Crystallogr D 65, 510–512. 19390156 10.1107/S0907444909007835

[54] Holm L & Rosenstrom P (2010) Dali server: conservation mapping in 3D. Nucleic Acids Res 38, 545–549. 10.1093/nar/gkq366 PMC2896194 20457744

[55] Sievers F , Wilm A , Dineen DG , Gibson TJ , Karplus K , Li W , Lopez R , McWilliam H , Remmert M , Söding J *et al* (2011) Fast, scalable generation of high‐quality protein multiple sequence alignments using Clustal Omega. Mol Syst Biol 7, 539. 21988835 10.1038/msb.2011.75 PMC3261699

[56] Tommasi R , Brown DG , Walkup GK , Manchester JI & Miller AA (2015) ESKAPEing the labyrinth of antibacterial discovery. Nat Rev Drug Dis 14, 529–542. 10.1038/nrd4572 26139286

[57] Hunter WN (2009) Structure‐based ligand design and the promise held for antiprotozoan drug discovery. J Biol Chem 284, 11749–11753. 19103598 10.1074/jbc.R800072200 PMC2673241

